# Computational cluster validation for microarray data analysis: experimental assessment of Clest, Consensus Clustering, Figure of Merit, Gap Statistics and Model Explorer

**DOI:** 10.1186/1471-2105-9-462

**Published:** 2008-10-29

**Authors:** Raffaele Giancarlo, Davide Scaturro, Filippo Utro

**Affiliations:** 1 Dipartimento di Matematica ed Applicazioni, Universitá di Palermo, Via Archirafi 34, 90123 Palermo, Italy

## Abstract

**Background:**

Inferring cluster structure in microarray datasets is a fundamental task for the so-called -omic sciences. It is also a fundamental question in Statistics, Data Analysis and Classification, in particular with regard to the prediction of the number of clusters in a dataset, usually established via internal validation measures. Despite the wealth of internal measures available in the literature, new ones have been recently proposed, some of them specifically for microarray data.

**Results:**

We consider five such measures: Clest, Consensus (Consensus Clustering), FOM (Figure of Merit), Gap (Gap Statistics) and ME (Model Explorer), in addition to the classic WCSS (Within Cluster Sum-of-Squares) and KL (Krzanowski and Lai index). We perform extensive experiments on six benchmark microarray datasets, using both Hierarchical and K-means clustering algorithms, and we provide an analysis assessing both the intrinsic ability of a measure to predict the correct number of clusters in a dataset and its merit relative to the other measures. We pay particular attention both to precision and speed. Moreover, we also provide various fast approximation algorithms for the computation of Gap, FOM and WCSS. The main result is a hierarchy of those measures in terms of precision and speed, highlighting some of their merits and limitations not reported before in the literature.

**Conclusion:**

Based on our analysis, we draw several conclusions for the use of those internal measures on microarray data. We report the main ones. Consensus is by far the best performer in terms of predictive power and remarkably algorithm-independent. Unfortunately, on large datasets, it may be of no use because of its non-trivial computer time demand (weeks on a state of the art PC). FOM is the second best performer although, quite surprisingly, it may not be competitive in this scenario: it has essentially the same predictive power of WCSS but it is from 6 to 100 times slower in time, depending on the dataset. The approximation algorithms for the computation of FOM, Gap and WCSS perform very well, i.e., they are faster while still granting a very close approximation of FOM and WCSS. The approximation algorithm for the computation of Gap deserves to be singled-out since it has a predictive power far better than Gap, it is competitive with the other measures, but it is at least two order of magnitude faster in time with respect to Gap. Another important novel conclusion that can be drawn from our analysis is that all the measures we have considered show severe limitations on large datasets, either due to computational demand (Consensus, as already mentioned, Clest and Gap) or to lack of precision (all of the other measures, including their approximations). The software and datasets are available under the GNU GPL on the supplementary material web page.

## Background

The recent advent of high density arrays of oligonucleotides and cDNAs has had a deep impact on biological and medical research. Indeed, the new technology enables the acquisition of data that is proving to be fundamental in many areas of the biological sciences, ranging from the understanding of complex biological systems to clinical diagnosis (see for instance the Stanford Microarray Database [1]).

Due to the large number of genes involved in each experiment, cluster analysis is a very useful exploratory technique for identifying genes that exhibit similar expression patterns, which may highlight groups of functionally related genes. This leads, in turn, to two well established and rich research areas. One deals with the design of new clustering algorithms and the other with the design of new internal validation measures that should assess the biological relevance of the clustering solutions found. Despite the vast amount of knowledge available in those two areas in the general data mining literature [2-9], gene expression data provide unique challenges, in particular with respect to internal validation measures. Indeed, they must predict how many clusters are really present in a dataset, an already difficult task, made even worse by the fact that the estimation must be sensible enough to capture the inherent biological structure of functionally related genes. Despite their potentially important role, both the use of classic internal validation measures and the design of new ones, specific for microarray data, do not seem to have great prominence in bioinformatics, where attention is mostly given to clustering algorithms. The excellent survey by Handl et al. [10] is a big step forward in making the study of those techniques a central part of both research and practice in bioinformatics, since it provides both a technical presentation as well as valuable general guidelines about their use for post-genomic data analysis. Although much remains to be done, it is, nevertheless, an initial step.

For instance, in the general data mining literature, there are several studies, e.g., [11], aimed at establishing the intrinsic, as well as the relative, merit of a measure. To this end, the two relevant questions are:

(A) What is the precision of a measure, i.e., its ability to predict the correct number of clusters in a data set? That is usually established by comparing the number of clusters predicted by the measure against the number of clusters in the gold solution of several datasets, the gold solution being a partition of the dataset in classes that can be trusted to be correct, i.e., distinct groups of functionally related genes. A more precise explanation of the meaning of gold solution in our setting is provided when we present the datasets used for our experiments in the Results and Discussion section.

(B) Among a collection of measures, which is more accurate, less algorithm-dependent, etc.,?. Precision versus the use of computational resources, primarily execution time, would be an important discriminating factor.

Although those classic studies are also of great relevance for bioinformatics, there is an acute need for analogous studies conducted on internal measures introduced recently and specifically designed for analysis of microarray data. We address both types of questions for several particularly prominent such measures, characterized by the fact that, for their prediction, they make use of nothing more than the dataset available: Clest [12], Consensus [13], FOM [14] Gap [15] and ME [16]. Because of their simplicity and computational efficiency, we also study WCSS [6] and KL [17]. The heuristic method supporting the use of WCSS as an internal measure has roots in the statistics community folklore [15].

Initial studies of the mentioned measures, in connection with both Questions (A) and (B), have been done, primarily, in the papers in which they were originally proposed. Our study carries further those studies by providing more focused information about using those measures for the analysis of gene expression data. For Question (A), our analysis provides further insights into the properties of the mentioned measures, with particular attention to time. For Question (B), we provide the first comparative analysis involving all of those measures that accounts for both precision and time. This is particularly relevant in regard to the "resampling-based" methods, i.e., Clest, Consensus and ME. In fact, (1) those three measures are excellent representatives of methods in the class (see Handl et al. and [18,19]); (2) Dudoit and Fridlyand mention that it would be desirable to relate Clest and ME but no comparison seems to be available in the literature; (3) although it is quite common to include Clest and Gap in comparative analysis for novel measures, Consensus is hardly considered, although our experiments show that it should definitely be included. Based on our analysis, we add to the state of the art valuable guidelines for the choice of which of the seven measures to use for microarray data analysis. Moreover, we also provide, as an additional contribution for FOM, Gap and WCSS, several good and fast approximation algorithms, i.e., the new algorithms have the same predictive power of the mentioned measures, while being faster. Particularly relevant is the approximation algorithm we propose for Gap. It is at least two orders of magnitude faster than the original measure and with a better prediction power.

## Results and discussion

### Experimental setup

#### Data sets

Technically speaking, a gold solution for a dataset is a partition of the data in a number of classes known *a priori*. Membership in a class is established by assigning the appropriate class label to each element. In less formal terms, the partition of the dataset in classes is based on external knowledge that leaves no ambiguity on the actual number of classes and on the membership of elements to classes. Although there exist real microarray datasets for which such an *a priori *division is known, in a few previous studies of relevance here, a more relaxed criterion has been adopted to allow also datasets with high quality partitions that have been inferred by analyzing the data, i.e., by the use of internal knowledge via data analysis tools such as clustering algorithms. In strict technical terms, there is a difference between the two types of "gold solutions". For their datasets, Dudoit and Fridlyand elegantly make clear that difference and we closely follow their approach here.

Each dataset is a matrix, in which each row corresponds to an element to be clustered and each column to an experimental condition. The six datasets, together with the acronyms used in this paper, are reported next. For conciseness, we mention only some relevant facts about them. The interested reader can find additional information in Handl et al., for the Leukemia dataset, in Dudoit and Fridlyand for the Lymphoma and NCI60 datasets and in Di Gesú et al. [20], for the remaining ones. In all of the referenced papers, the datasets were used for validation studies. Moreover, in those papers, the interested reader can find additional pointers to validation studies using the same datasets.

##### CNS Rat

The dataset gives the expression levels of 112 genes during rat central nervous system development. It is a 112 × 17 data matrix studied by Wen et al. [21]. There are no *a priori *known classes for this dataset, but the analysis by Wen et al. suggests a partition of the genes into six classes, four of which are composed of biologically, functionally-related, genes. We take that to be the gold solution, which is the same one used for the validation of FOM.

##### Leukemia

The dataset is the one used by Handl et al. in their survey of computational cluster validation to illustrate the use of some measures. It is a 38 × 100 data matrix, where each row corresponds to a patient with acute leukemia and each column to a gene. For this dataset, there is an *a priori *partition into three classes and we take that as the gold solution.

##### Lymphoma

The dataset comes from the study of Alizadeh et al. [22] on the three most common adult lymphoma tumors. It is an 80 × 100 matrix, where each row corresponds to a tissue sample and each column to a gene. There is an *a priori *partition into three classes and we take that as the gold solution. The dataset has been obtained from the original microarray experiments as described by Dudoit and Fridlyand.

##### NCI60

This dataset originates from a microarray study in gene expression variation among the sixty cell lines of National Cancer Institute anti-cancer drug screen [23]. It is a 57 × 200 data matrix, where each row corresponds to a cell line and each column to a gene. There is an *a priori *partition of the dataset into eight classes and we take that as the gold solution. The dataset has been obtained from the original microarray experiments as described by Dudoit and Fridlyand.

##### Yeast

The dataset is part of that studied by Spellman et al. [24] and it is a 698 × 72 data matrix. There are no *a priori *known classes for this dataset, but the analysis by Spellman et al. suggests a partition of the genes into five functionally-related classes. We take that to be the gold solution, which has been used by Shamir amd Sharan for a case study on performance of clustering algorithms [25].

##### PBM

The dataset contains 2329 cDNAs with a fingerprint of 139 oligos. This gives a 2329 × 139 data matrix. According to Hartuv et al. [26], the cDNAs in the dataset originated from 18 distinct genes, i.e., the *a priori *classes are known. The partition of the dataset into 18 groups was obtained by lab experiments at Novartis in Vienna. Following that study, we take those classes and the class labels assigned to the elements as the gold solution. It was used by Hartuv et al. to test their clustering algorithm.

#### Clustering algorithms and their stability

We use a suite of clustering algorithms. Among the hierarchical methods [27] Hier-A (Average Link), Hier-C (Complete Link), and Hier-S (Single Link). Moreover, we use K-means [27], both in the version that starts the clustering from a random partition of the data and in the version where it takes as part of the input an initial partition produced by one of the chosen hierarchical methods. The acronyms of those versions are K-means-R, K-means-A, K-means-C and K-means-S, respectively.

We also point out that K-means-R is a randomized algorithm that may provide different answers on the same input dataset. That might make the values of many of the measures we are studying depend critically on the particular execution of the algorithm. Such a dependence is important for WCSS, KL and FOM. For those measures and their approximations, we have repeated five times the computation of the relevant curves, on all datasets, with K-means-R. We observed only negligible differences from run to run. Therefore, in what follows, all reported results refer to a single run of the algorithms, except for the cases in which an explicit Monte Carlo simulation is required.

#### Similarity/Distance Functions

All of our algorithms use Euclidean distance in order to assess similarity of single elements to be clustered. Such a choice is natural and conservative, as we now explain. It places all algorithms in the same position without introducing biases due to distance function performance, rather than to the algorithm. Moreover, time course data have been properly standardized (mean equal to zero and variance equal to one), so that Euclidean distance would not be penalized on those data. This is standard procedure, e.g., [14], for those data. The results we obtain are conservative since, assuming that one has a provably much better similarity/distance function, one can only hope to get better estimates than ours (else the used distance function is not better than Euclidean distance after all). As it is clear from the upcoming discussion and conclusions, such better estimates will cause no dramatic change in the general picture of our findings. The choice is also natural, in view of the debate regarding the identification of a proper similarity/distance function for clustering gene expression data and the number of such measures available. The state of the art as well some relevant progress in the identification of such measure is well presented in [28].

#### Hardware

All experiments for the assessment of the precision of each measure were performed in part on several state-of-the-art PCs and in part on a 64-bit AMD Athlon 2.2 GHz bi-processor with 1 GB of main memory running Windows Server 2003. All the timing experiments reported were performed on the bi-processor, using one processor per run. The usage of different machines for the experimentation was deemed necessary in order to complete the full set of experiments in a reasonable amount of time. Indeed, as detailed later, some measures require weeks to complete execution on Yeast and PBM, the two largest datasets we have used. We also point out that all the Operating Systems supervising the computations have a 32 bits precision.

### Question (A)-Intrinsic precision of the internal measures

In this section we present experiments with the aim to shed some light on Question (A). As discussed in the Methods section, for most measures, the prediction of the "optimal" number k* of clusters is based on the visual inspection of curves and histograms. For conciseness, we provide all the relevant material in the supplementary material web site [29] (Figures section). Here we limit ourselves to produce summary tables, based on our analysis of the relevant curves and experiments. We report a separate table for each measure, (see Tables 1, 2, 3, 4, 5, 6, 7, 8). We anticipate that the next subsection addresses the relative merits of each measure and a global summary table is reported, but only for the best performers. That is, for each measure, we report the experimental parameters (e.g., clustering algorithm) only if in that setting the prediction of k* has been reasonably close to the gold solution (at most an absolute value difference of one between the predicted number and the real number) in at least four of the six datasets we have used. Table 9 is a summary of those results. The reader interested mainly in a comparative analysis of the various measures may wish to skip this subsection.

**Table 1:** Results for WCSS and its approximations

	Precision						Timing			
	
	CNS Rat	Leukemia	NCI60	Lymphoma	Yeast	PBM	CNS Rat	Leukemia	NCI60	Limphoma
K-means-R	4	➌	3	8	④	-	2.4 × 10^3^	2.0 × 10^3^	8.4 × 10^3^	8.4 × 10^3^
K-means-A	4	➌	⑦	6	➎	-	2.3 × 10^3^	1.3 × 10^3^	5.4 × 10^3^	5.8 × 10^3^
K-means-C	⑤	➌	➑	8	④	-	1.7 × 10^3^	1.3 × 10^3^	5.0 × 10^3^	4.0 × 10^3^
K-means-S	3	④	⑦	8	24	-	2.6 × 10^3^	1.6 × 10^3^	7.3 × 10^3^	7.4 × 10^3^
R-R0	⑤	④	⑨	➌	④	-	1.2 × 10^3^	8.0 × 10^2^	4.1 × 10^3^	3.0 × 10^3^
R-R5	➏	5	⑨	5	④	-	1.2 × 10^3^	8.0 × 10^2^	4.6 × 10^3^	3.2 × 10^3^
R-R2	⑦	5	15	④	④	-	1.3 × 10^3^	8.0 × 10^2^	5.3 × 10^3^	3.2 × 10^3^
Hier-A	10	➌	3	6	➎	-	1.1 × 10^3^	4.0 × 10^2^	2.1 × 10^3^	1.9 × 10^3^
Hier-C	10	➌	⑦	8	9	-	7.0 × 10^2^	4.0 × 10^2^	1.7 × 10^3^	1.4 × 10^3^
Hier-S	8	10	⑦	9	-	-	2.6 × 10^3^	6.0 × 10^2^	3.2 × 10^3^	3.8 × 10^3^
**Gold solution**	**6**	**3**	**8**	**3**	**5**	**18**	-	-	-	-

A summary of the results for WCSS on all algorithms and on all datasets. The columns under the label precision indicate the number of clusters predicted by WCSS, while the remaining four indicate the timing in milliseconds for the execution of the corresponding experiment. Cells with a dash indicate that WCSS did not give any useful indication.

**Table 2:** Results for KL

	Precision						Timing			
	
	CNS Rat	Leukemia	NCI60	Lymphoma	Yeast	PBM	CNS Rat	Leukemia	NCI60	Limphoma
K-means-R	4	27	3	22	29	24	2.7 × 10^3^	3.4 × 10^3^	9.3 × 10^3^	9.0 × 10^3^
K-means-A	25	➌	3	②	7	16	2.3 × 10^3^	2.4 × 10^3^	5.7 × 10^3^	6.2 × 10^3^
K-means-C	2	➌	⑦	②	26	24	3.0 × 10^3^	2.6 × 10^3^	5.0 × 10^3^	5.8 × 10^3^
K-means-S	4	④	12	8	13	16	4.0 × 10^3^	2.9 × 10^3^	8.0 × 10^3^	8.5 × 10^3^
Hier-A	⑦	➌	3	②	17	12	1.9 × 10^3^	6.0 × 10^2^	2.1 × 10^3^	2.5 × 10^3^
Hier-C	10	➌	2	②	16	15	1.6 × 10^3^	1.1 × 10^3^	2.5 × 10^3^	2.1 × 10^3^
Hier-S	21	7	⑦	9	15	25	3.4 × 10^3^	1.3 × 10^3^	3.7 × 10^3^	4.9 × 10^3^
**Gold solution**	**6**	**3**	**8**	**3**	**5**	**18**	-	-	-	-

A summary of the results for KL on all algorithms and on all datasets. The columns under the label precision indicate the number of clusters predicted by KL, while the remaining four indicate the timing in milliseconds for the execution of the corresponding experiment.

**Table 3:** Results for G-Gap

	Precision						Timing			
	
	CNS Rat	Leukemia	NCI60	Lymphoma	Yeast	PBM	CNS Rat	Leukemia	NCI60	Limphoma
K-means-R	⑦	➌	4	④	⑥	5	2.4 × 10^3^	2.0 × 10^3^	8.3 × 10^3^	8.4 × 10^3^
K-means-A	4	➌	1	②	⑥	4	2.3 × 10^3^	1.3 × 10^3^	5.3 × 10^3^	5.8 × 10^3^
K-means-C	⑤	➌	2	8	⑥	5	1.7 × 10^3^	1.3 × 10^3^	5.0 × 10^3^	4.0 × 10^3^
K-means-S	3	➌	1	1	1	1	2.6 × 10^3^	1.6 × 10^3^	7.3 × 10^3^	7.4 × 10^3^
R-R0	2	7	2	④	➎	4	1.2 × 10^3^	8.0 × 10^2^	4.0 × 10^3^	3.0 × 10^3^
R-R5	⑤	④	2	②	④	6	1.2 × 10^3^	8.0 × 10^2^	4.5 × 10^3^	3.2 × 10^3^
R-R2	3	②	2	②	➎	6	1.3 × 10^3^	8.0 × 10^2^	5.2 × 10^3^	3.2 × 10^3^
Hier-A	⑦	➌	1	②	3	1	1.1 × 10^3^	4.0 × 10^2^	2.0 × 10^3^	1.9 × 10^3^
Hier-C	⑦	➌	2	②	7	4	7.0 × 10^2^	4.0 × 10^2^	1.7 × 10^3^	1.4 × 10^3^
Hier-S	⑤	➌	1	1	1	5	2.6 × 10^3^	6.0 × 10^2^	3.2 × 10^3^	3.8 × 10^3^
**Gold solution**	**6**	**3**	**8**	**3**	**5**	**18**	-	-	-	-

A summary of the results of G-Gap, on all algorithms and on all datasets. The columns under the label precision indicate the number of clusters predicted by the measure, while the remaining four indicate the timing in milliseconds for the execution of the corresponding experiment.

**Table 4:** Results for Clest

	Precision					Timing
	
	CNS Rat	Leukemia	NCI60	Lymphoma	Yeast	CNS Rat
Clest-FM-K-means-R	8	④	➑	②	④	1.2 × 10^6^
Clest-FM-K-means-A	18	7	12	15	13	1.4 × 10^6^
Clest-FM-K-means-C	12	5	12	11	④	1.5 × 10^6^
Clest-FM-K-means-S	24	8	13	15	1	1.8 × 10^6^
Clest-FM-Hier-A	10	6	10	13	24	1.1 × 10^6^
Clest-FM-Hier-C	10	④	⑨	15	8	1.1 × 10^6^
Clest-FM-Hier-S	20	10	15	15	1	1.1 × 10^6^
Clest-Adj-K-means-R	⑤	④	3	②	2	1.1 × 10^6^
Clest-Adj-K-means-A	12	➌	3	②	➎	1.4 × 10^6^
Clest-Adj-K-means-C	9	②	2	②	④	1.4 × 10^6^
Clest-Adj-K-means-S	20	6	13	6	10	1.8 × 10^6^
Clest-Adj-Hier-A	13	➌	3	②	11	1.1 × 10^6^
Clest-Adj-Hier-C	9	④	2	②	④	1.1 × 10^6^
Clest-Adj-Hier-S	4	7	⑨	7	26	1.1 × 10^6^
Clest-F-K-means-R	➏	➌	15	②	④	1.2 × 10^6^
Clest-F-K-means-A	8	6	10	14	11	1.4 × 10^6^
Clest-F-K-means-C	9	5	12	➌	④	1.5 × 10^6^
Clest-F-K-means-S	21	10	15	15	1	1.8 × 10^6^
Clest-F-Hier-A	⑦	7	10	15	27	1.1 × 10^6^
Clest-F-Hier-C	9	➌	13	➌	➎	1.1 × 10^6^
Clest-F-Hier-S	28	10	15	15	1	1.1 × 10^6^
**Gold solution**	**6**	**3**	**8**	**3**	**5**	-

A summary of the results for Clest on all algorithms and the first four datasets, with use of three external indexes. The columns under the label precision indicate the number of clusters predicted by Clest, while the remaining one indicates the timing in milliseconds for the execution of the corresponding experiment. For PBM, the experiments were terminated due to their high computational demand (weeks to complete). Therefore, the resulting column is omitted from the table. For the Leukemia, NCI60 and Lymphoma datasets, the timing experiments are not reported because incomparable with those of CNS Rat and of the other measures. The corresponding columns are eliminated from the table.

**Table 5:** Results for Consensus

	Precision					Timing			
	
	CNS Rat	Leukemia	NCI60	Lymphoma	Yeast	CNS Rat	Leukemia	NCI60	Lymphoma
K-means-R	➏	④	⑦	➌	⑥	1.0 × 10^6^	1.3 × 10^6^	3.4 × 10^6^	3.0 × 10^6^
K-means-A	⑦	➌	➑	➌	⑥	1.3 × 10^6^	1.6 × 10^6^	3.0 × 10^6^	2.6 × 10^6^
K-means-C	➏	➌	➑	④	⑥	1.3 × 10^6^	1.8 × 10^6^	2.9 × 10^6^	2.6 × 10^6^
K-means-S	⑦	④	10	②	⑥	1.5 × 10^6^	1.8 × 10^6^	3.2 × 10^6^	2.8 × 10^6^
Hier-A	⑦	➌	➑	➌	➎	9.2 × 10^5^	7.9 × 10^5^	2.0 × 10^6^	1.9 × 10^6^
Hier-C	➏	④	➑	5	⑥	8.7 × 10^5^	6.9 × 10^5^	2.0 × 10^6^	2.0 × 10^6^
Hier-S	2	8	10	➌	10	9.4 × 10^5^	8.0 × 10^5^	2.0 × 10^6^	1.7 × 10^6^
**Gold solution**	**6**	**3**	**8**	**3**	**5**	-	-	-	-

A summary of the results for Consensus on all algorithms and the first five datasets. The columns under the label precision indicate the number of clusters predicted by Consensus, while the remaining four indicate the timing in milliseconds for the execution of the corresponding experiment. For PBM, the experiments were terminated due to their high computational demand and the corresponding column has been removed from the table.

**Table 6:** Results for FOM

	Precision						Timing			
	
	CNS Rat	Leukemia	NCI60	Lymphoma	Yeast	PBM	CNS Rat	Leukemia	NCI60	Lymphoma
K-means-R	⑦	➌	6	9	④	-	2.9 × 10^4^	1.9 × 10^5^	1.3 × 10^6^	6.7 × 10^5^
K-means-A	⑦	➌	6	6	④	-	2.2 × 10^4^	9.3 × 10^4^	5.5 × 10^5^	2.7 × 10^5^
K-means-C	⑦	8	➑	④	④	-	1.9 × 10^4^	9.4 × 10^4^	5.5 × 10^5^	2.6 × 10^5^
K-means-S	➏	➌	➑	8	④	-	2.9 × 10^4^	1.0 × 10^5^	7.1 × 10^5^	3.6 × 10^5^
R-R0	10	5	⑦	④	7	-	2.6 × 10^3^	3.1 × 10^4^	1.7 × 10^5^	5.3 × 10^4^
R-R5	➏	➌	⑦	5	➎	-	3.9 × 10^3^	3.7 × 10^4^	2.1 × 10^5^	7.6 × 10^4^
R-R2	8	5	➑	5	➎	-	3.4 × 10^3^	3.8 × 10^4^	2.2 × 10^5^	7.2 × 10^4^
Hier-A	⑦	➌	⑦	6	⑥	-	1.6 × 10^3^	7.5 × 10^3^	5.1 × 10^4^	1.8 × 10^4^
Hier-C	10	④	⑦	7	➎	-	1.6 × 10^3^	7.7 × 10^3^	4.5 × 10^4^	1.8 × 10^4^
Hier-S	3	7	⑦	9	-	-	1.6 × 10^3^	7.4 × 10^3^	4.9 × 10^5^	1.7 × 10^4^
**Gold solution**	**6**	**3**	**8**	**3**	**5**	**18**	-	-	-	-

A summary of the results for FOM on all algorithms and on all datasets. The columns under the label precision indicate the number of clusters predicted by FOM, while the remaining four indicate the timing in milliseconds for the execution of the corresponding experiment. Cells with a dash indicate that FOM did not give any useful indication.

**Table 7:** Results for G-FOM

	Precision						Timing			
	
	CNS Rat	Leukemia	NCI60	Lymphoma	Yeast	PBM	CNS Rat	Leukemia	NCI60	Lymphoma
K-means-R	⑦	5	6	8	⑥	7	2.9 × 10^4^	1.9 × 10^5^	1.3 × 10^6^	6.7 × 10^5^
K-means-A	2	➌	⑦	②	⑥	6	2.2 × 10^4^	9.3 × 10^4^	5.5 × 10^5^	2.7 × 10^5^
K-means-C	2	④	2	④	7	6	1.9 × 10^4^	9.4 × 10^4^	5.5 × 10^5^	2.6 × 10^5^
K-means-S	3	5	2	②	⑥	8	2.9 × 10^4^	1.0 × 10^5^	7.1 × 10^5^	3.6 × 10^5^
R-R0	2	7	2	5	7	4	2.6 × 10^3^	3.1 × 10^4^	1.7 × 10^5^	5.3 × 10^4^
R-R5	4	④	2	6	⑥	4	3.9 × 10^3^	3.7 × 10^4^	2.1 × 10^5^	7.6 × 10^4^
R-R2	⑦	5	2	5	8	4	3.4 × 10^3^	3.8 × 10^4^	2.2 × 10^5^	7.2 × 10^4^
Hier-A	3	➌	⑦	②	8	2	1.6 × 10^3^	7.5 × 10^3^	5.1 × 10^4^	1.8 × 10^4^
Hier-C	10	④	2	④	8	2	1.6 × 10^3^	7.7 × 10^3^	4.5 × 10^4^	1.8 × 10^4^
Hier-S	⑦	②	2	②	2	2	1.6 × 10^3^	7.4 × 10^3^	4.9 × 10^5^	1.7 × 10^4^
**Gold solution**	**6**	**3**	**8**	**3**	**5**	**18**	-	-	-	-

A summary of the results for G-FOM on all algorithms and on all datasets. The columns under the label precision indicate the number of clusters predicted by G-FOM, while the remaining four indicate the timing in milliseconds for the execution of the corresponding experiment. Cells with a dash indicate that G-FOM did not give any useful indication.

**Table 8:** Results for DIFF-FOM

	Precision						Timing			
	
	CNS Rat	Leukemia	NCI60	Lymphoma	Yeast	PBM	CNS Rat	Leukemia	NCI60	Lymphoma
K-means-R	4	➌	4	➌	3	4	2.9 × 10^4^	1.9 × 10^5^	1.3 × 10^6^	6.7 × 10^5^
K-means-A	⑦	➌	3	6	3	8	2.2 × 10^4^	9.3 × 10^4^	5.5 × 10^5^	2.7 × 10^5^
K-means-C	⑦	➌	⑦	④	3	5	1.9 × 10^4^	9.4 × 10^4^	5.5 × 10^5^	2.6 × 10^5^
K-means-S	⑦	➌	12	8	3	10	2.9 × 10^4^	1.0 × 10^5^	7.1 × 10^5^	3.6 × 10^5^
R-R0	10	④	17	④	3	3	2.6 × 10^3^	3.1 × 10^4^	1.7 × 10^5^	5.3 × 10^4^
R-R5	4	➌	11	➌	3	4	3.9 × 10^3^	3.7 × 10^4^	2.1 × 10^5^	7.6 × 10^4^
R-R2	⑦	➌	17	➌	3	7	3.4 × 10^3^	3.8 × 10^4^	2.2 × 10^5^	7.2 × 10^4^
Hier-A	⑦	➌	3	6	3	25	1.6 × 10^3^	7.5 × 10^3^	5.1 × 10^4^	1.8 × 10^4^
Hier-C	9	➌	⑦	7	3	7	1.6 × 10^3^	7.7 × 10^3^	4.5 × 10^4^	1.8 × 10^4^
Hier-S	20	7	22	9	7	20	1.6 × 10^3^	7.4 × 10^3^	4.9 × 10^5^	1.7 × 10^4^
**Gold solution**	**6**	**3**	**8**	**3**	**5**	**18**	-	-	-	-

A summary of the results for DIFF-FOM on all algorithms and on all datasets. The columns under the label precision indicate the number of clusters predicted by DIFF-FOM, while the remaining four indicate the timing in milliseconds for the execution of the corresponding experiment. Cells with a dash indicate that DIFF-FOM did not give any useful indication.

**Table 9:** Summary of results for the best performing measures

	Precision					Timing			
	
	CNS Rat	Leukemia	NCI60	Lymphoma	Yeast	CNS Rat	Leukemia	NCI60	Lymphoma
WCSS-K-means-C	⑤	➌	➑	8	④	1.7 × 10^3^	1.3 × 10^3^	5.0 × 10^3^	4.0 × 10^3^
WCSS-R-R0	⑤	④	⑨	➑	④	1.2 × 10^3^	8.0 × 10^2^	4.1 × 10^3^	3.0 × 10^3^
G-Gap-K-means-R	⑦	➌	4	④	⑥	2.4 × 10^3^	2.0 × 10^3^	8.3 × 10^4^	8.4 × 10^3^
G-Gap-R-R5	⑤	④	2	②	④	1.2 × 10^3^	8.0 × 10^2^	4.5 × 10^4^	3.2 × 10^3^
FOM-K-means-C	⑦	8	➑	④	④	1.9 × 10^4^	9.4 × 10^4^	5.5 × 10^5^	2.6 × 10^5^
FOM-K-means-S	➏	➌	➑	8	④	2.9 × 10^4^	1.0 × 10^5^	7.1 × 10^5^	3.6 × 10^5^
FOM-R-R5	➏	➌	⑦	5	➎	3.9 × 10^3^	3.7 × 10^4^	2.1 × 10^5^	7.6 × 10^4^
FOM-Hier-A	⑦	➌	⑦	6	⑥	1.6 × 10^3^	7.5 × 10^3^	5.1 × 10^4^	1.8 × 10^4^
DIFF-FOM-K-means-C	⑦	➌	⑦	④	3	1.9 × 10^4^	9.4 × 10^4^	5.5 × 10^5^	2.6 × 10^5^
Clest-F-K-means-R	➏	➌	15	②	④	1.2 × 10^6^	-	-	-
Clest-FM-K-means-R	8	④	➑	②	④	1.2 × 10^6^	-	-	-
Consensus-K-means-R	➏	④	⑦	➌	⑥	1.0 × 10^6^	1.3 × 10^6^	3.4 × 10^6^	3.0 × 10^6^
Consensus-K-means-A	⑦	➌	➑	➌	⑥	1.3 × 10^6^	1.6 × 10^6^	3.0 × 10^6^	2.6 × 10^6^
Consensus-K-means-C	➏	➌	➑	④	⑥	1.3 × 10^6^	1.8 × 10^6^	2.9 × 10^6^	2.6 × 10^6^
Consensus-K-means-S	⑦	④	10	②	⑥	1.5 × 10^6^	1.8 × 10^6^	3.2 × 10^6^	2.8 × 10^6^
Consensus-Hier-A	⑦	➌	➑	➌	➎	9.2 × 10^5^	7.9 × 10^5^	2.0 × 10^6^	1.9 × 10^6^
Consensus-Hier-C	➏	④	➑	5	⑥	8.7 × 10^5^	6.9 × 10^5^	2.0 × 10^6^	2.0 × 10^6^
**Gold solution**	**6**	**3**	**8**	**3**	**5**	-	-	-	-

A summary of the best performances obtained by each measure. The PBM dataset has been excluded because no measure gave useful information about its cluster structure.

Moreover, in what follows, for each cell in a table displaying precision results, a number in a circle with a black background indicates a prediction in agreement with the number of classes in the dataset, while a number in a circle with a white background indicates a prediction that differs, in absolute value, by 1 from the number of classes in the dataset; when the prediction is one cluster, i.e. Gap statistics, this symbol rule is not applied because the prediction means no cluster structure in the data; a number not in a circle indicates the remaining predictions. As detailed in each table, cells with a dash indicate that either the experiment was stopped, because of its high computational demand, or that the measure gives no useful indication. The timing results are reported only on the four smallest datasets. Indeed, for Yeast and PBM, the computational demand is such on some measures that either they had to be stopped or they took weeks to complete. For those two datasets, the experiments we report were done using more than one machine.

#### WCSS and its approximations

For each algorithm, each of the WCSS approximations (denoted WCSS-R-R0, WCSS-R-R2, WCSS-R-R5, respectively), and each dataset, we have computed WCSS for a number of cluster values in the range [2,30]. The relevant plots are in the Figures section at the supplementary material web site: Fig. S1 for the K-means algorithms and WCSS approximations and Fig. S2 for the hierarchical algorithms.

As outlined in the Methods section, given the relevant WCSS curve, k* is predicted as the abscissa closest to the "knee" in that curve. The values resulting from the application of this methodology to the relevant plots are reported in Table 1 together with timing results for the relevant datasets.

We have that WCSS performs well with K-means-C and K-means-A, on the first five datasets, while it gives no reasonably correct indication on PBM. It is a poor performer with the other clustering algorithms. Those facts give strong indication that WCSS is algorithm-dependent. Finally, the failure of WCSS, with all algorithms, to give a good prediction for PBM indicates that WCSS may not be of any use on large datasets having a large number of clusters.

As for its approximations, it is evident that they perform better than the original WCSS curve (obtained via all other clustering algorithms we have experimented with). That is, they are among the best performers in Table 1. Moreover, depending on the dataset, they are from a few times to an order of magnitude faster than the K-means algorithms.

Overall, the best performers are K-means-C and WCSS-R-R0. The relative results are reported in Table 9 for comparison with the performance of the other measures.

#### KL

Following the same experimental set-up of WCSS, we have computed the KL measure, for each dataset and each algorithm. The results, summarized in Table 2, are rather disappointing: the measure provides some reliable indication, accross algorithms, only on the Leukemia and the Lymphoma datasets. Due to such a poor performance, no results are reported in Table 9 for comparison with the performance of the other measures.

#### Gap and its geometric approximation

For each dataset and each clustering algorithm, we compute three versions of Gap, namely Gap-Ps, Gap-Pc and Gap-Pr, for a number of cluster values in the range [1,30]. Gap-Ps uses the Poisson null model, Gap-Pc the Poisson null model aligned with the principal components of the data while Gap-Pr uses the permutational null model (see Methods section). For each of them, we perform a Monte Carlo simulation, 20 steps, in which the measure returns an estimated number of clusters for each step. Each simulation step is based on the generation of 10 data matrices from the null model used by the measure. At the end of each Monte Carlo simulation, the number with the majority of estimates is taken as the predicted number of clusters. Occasionally, there are ties and we report both numbers. The relevant histograms are displayed at the supplementary material web site (Figures section): Figs. S3-S8 for Gap-Ps, Figs. S9-S13 for Gap-Pc and Figs. S14-S19 for Gap-Pr. The results are summarized in Table T1 at the supplementary material web page (Tables section). For PBM and Gap-Pc, each experiment was terminated after a week, since no substantial progress was being made towards its completion.

**Figure 1:**
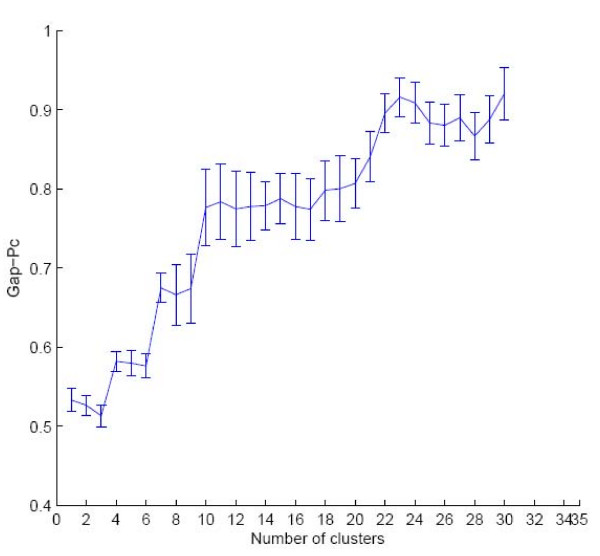
**A Gap Curve**. The Gap-Pc curve on the Leukemia dataset, with use of the Hier-S algorithm. At each point, error bars indicate the variation of the curve accross simulations. The curve shows a first maximum at *k *= 1, yielding a prediction of k* = 1, the next maximum is at *k *= 4, which is very close to the number of classes k* = 3.

The results for Gap are somewhat disappointing, as Table T1 shows, and therefore given only for completeness at the supplementary material web site. However, a few comments are in order, the first one regarding the null models. Tibshirani et al. find experimentally that, on simulated data, Gap-Pc is the clear winner over Gap-Ps (they did not consider Gap-Pr). Our results show that, as the dataset size increases, Gap-Pc incurs into a severe time performance degradation, due to the repeated data transformation step. Moreover, on the smaller datasets, no null model seems to have the edge. Some of the results are also somewhat puzzling. In particular, although the datasets have cluster structure, many algorithms return an estimate of k* = 1, i.e., no cluster structure in the data. An analogous situation was reported by Monti et al. In their study, Gap-Ps returned k* = 1 on two artificial datasets. Fortunately, an analysis of the corresponding Gap curve showed that indeed the first maximum was at k* = 1 but a local maximum was also present at the correct number of classes, in each dataset. We have performed an analogous analysis of the relevant Gap curves to find that, in analogy with Monti et al., also in our case most curves show a local maximum at or very close to the number of classes in each dataset, following the maximum at k* = 1. An example curve is given in Fig. 1. From the above, one can conclude that inspection of the Gap curves and *domain knowledge *can greatly help in disambiguating the case k* = 1. We also report that experiments conducted by Dudoit and Fridlyand and, independently by Yan and Ye [30], show that Gap tends to overestimate the correct number of clusters, although this does not seem to be the case for our datasets and algorithms. The above considerations seem to suggest that the automatic rule for the prediction of k* based on Gap is rather weak.

As for G-Gap, the geometric approximation of Gap, we have computed, for each algorithm and each dataset, the corresponding WCSS curve and its approximations in the interval [1,30]. We have then applied the rule described in the Methods section to get the value of k*. The results are summarized in Table 3. As it is evident from the table, the overall performance of G-Gap is clearly superior to Gap, irrespective of the null model. Moreover, depending on the dataset, it is from two to three orders of magnitude faster. The best performers are K-means-R and WCSS-R-R0. The relative results are reported in Table 9 for comparison with the performance of the other measures.

#### Clest

For CNS Rat and Yeast and each clustering algorithm, we compute Clest for a number of cluster values in the range [2,30] while, for Leukemia, NCI60 and Lymphoma, the ranges [2,10], [2,15] and [2,15] are used, respectively, due to the small size of the datasets. Moreover, although experiments have been started with PBM, no substantial progress was made after a week of execution and, for each clustering algorithm, the corresponding experiment was terminated. Following the same experimental set-up of Dudoit and Fridlyand, for each cluster value *k*, we perform 20 resampling steps and 20 iterations. In each step, 66% of the rows of the data matrix are extracted, uniformly and at random, to create a learning set, to be given to the clustering algorithm to be clustered in *k *groups. As one of its input parameters, Clest requires the use of an external index *E *to establish the level of agreement between two partitions of a dataset. We use each of the following: the FM index [31], Adj (the Adjusted Rand index) [32] and F (the F-index) [33].

The results are summarized in Table 4, where the timing results for the Leukemia, NCI60 and Lymphoma datasets were excluded since the experiments were performed on a smaller interval of cluster values with respect to CNS Rat. This latter interval is the standard one we are using to make consistent comparisons across measures and algorithms.

The results show that Clest has severe time demand limitations on large datasets. It also seems to achieve a better performance, accross algorithms with Adj and F. Moreover, it is clearly algorithm-dependent, with K-means-R being the best performer with both FM and F. Those results are reported in Table 9 for comparison with the performance of the other measures.

#### ME

For each of the first five datasets and each clustering algorithm, we compute ME for a number of cluster values in the range [2,30]. Following the same experimental set-up of Ben-Hur et al., for each cluster value *k*, we perform 100 iterations. In each step, we compute two datasets to be given to the algorithm to be clustered in *k *groups. Each dataset is created by extracting uniformly and at random 80% of the rows. The prediction of k* is based on the plot of the corresponding histograms, as illustrated in the Methods section. As for the external indexes, we have used the same three used for Clest. The histograms obtained from such an experimentation are reported at the supplementary material web site in Figs. S20-S124. As for PBM, the computations were stopped because of their computational demand. A summary of the results is given in Table T2 at the supplementary material web site for completeness only. Indeed, the performance of ME was rather disappointing, with the exception of Leukemia and Lymphoma, accross algorithms and external indexes.

#### Consensus

For each of the first five datasets and each clustering algorithm, we compute Consensus for a number of cluster values in the range [2,30]. Following the same experimental set-up of Monti et al., for each cluster value *k*, we perform 500 resampling steps. In each step, 80% of the rows of the matrix are extracted uniformly and at random to create a new dataset, to be given to the clustering algorithm to be clustered in *k *groups. The prediction of k* is based on the plot of two curves, Δ(*k*) and Δ'(*k*), as a function of the number *k *of clusters. Both curves are defined in the Methods section. As suggested by Monti et al., the first curve is suitable for hierarchical algorithms while the second suits non-hierarchical ones. We did not experiment for PBM, since Consensus was very slow (execution on each algorithm was terminated after a week). Contrary to Monti et al. indication, we have computed the Δ(*k*) curve for all algorithms on the first five datasets, for reasons that will be self-evident shortly. The corresponding plots are available at the supplementary material web site (Figures section) as Figs. S125-S134. We have also computed the Δ(*k*) curve for the K-means algorithms, on the same datasets. Since those curves are nearly identical to the Δ(*k*) ones, they are omitted. In order to predict the number of clusters in the datasets, we have used, for all curves, the rule reported and explained in the Methods section: take as k* the abscissa corresponding to the smallest non-negative value where the curve starts to stabilize; that is, no big variation in the curve takes place from that point on. We performed such an analysis on the Δ(*k*) curves and the results are summarized in Table 5, together with the corresponding timing results.

As for the precision of Consensus, all algorithms perform well, except for Hier-S. The results also show that the Δ curve may be adequate for all algorithms. This contradicts the recommendation by Monti et al. and a brief explanation of the reason is given in the Methods section.

In conclusion, Consensus seems to be limited by time demand that makes it not applicable to large datasets. However, on small and medium sized datasets, it is remarkably precise across algorithms. In fact, except for Hier-S, the performance of Consensus is among the best and reported in Table 9 for comparison with the performance of the other measures.

#### FOM and its extensions and approximations

For each algorithm, each of the FOM approximations (denoted FOM-R-R0, FOM-R-R2, FOM-R-R5, respectively) and each dataset, we have followed the same methodology outlined for WCSS. The relevant plots are in Figs. S135-S136 at the supplementary material web site (Figures section). The values resulting from the application of this methodology to the relevant plots are reported in Table 6 together with timing results for the relevant datasets. Using the same experimental setting, we have also computed the k* predicted by G-FOM and DIFF-FOM, the extensions of FOM proposed here. The results are in Tables 7 and 8, respectively. As those results show, G-FOM did not perform as well as the other two. Moreover, both FOM and DIFF-FOM are algorithm-dependent and give no useful indication on large datasets. As for the approximations of FOM, i.e., FOM-R-R0, FOM-R-R2, FOM-R-R5, they compare very well with the K-means algorithms in terms of precision and they are an order of magnitude faster. The best performing methods, both for FOM and DIFF-FOM, are reported in Table 9 for comparison with the performance of the other measures.

### Question (B): relative merits of each measure

The discussion here refers to Table 9. It is evident that the K-means algorithms have superior performance with respect to the hierarchical ones, although Hier-A has an impressive and unmatched performance with Consensus. The approximation algorithms we have proposed for both WCSS and FOM are among the best performers. G-Gap and DIFF-FOM also guarantee, with a proper choice of algorithms, a good performance. In particular, G-Gap is orders of magnitude faster than Gap and more precise.

However, Consensus and FOM stand out as being the most stable across algorithms. In particular, Consensus has a remarkable stability performance accross algorithms and datasets.

For large datasets such as PBM, our experiments show that all the measures are severely limited due either to speed (Clest, Consensus, Gap-Pc) or to precision as well as speed (the others). In our view, this fact stresses even more the need for good data filtering and dimensionality reduction techniques since they may help reduce such datasets to sizes manageable by the measures we have studied.

It is also obvious that, when one takes computer time into account, there is a hierarchy of measures, with WCSS being the fastest and Consensus the slowest. We see from Table 9 that there is a natural division of methods in two groups: slow (Clest, Consensus, Gap) and fast (the other measures). Since there are at least two orders of magnitude of difference in time performance between the two groups, it seems reasonable to use one of the fast methods, for instance G-Gap, to limit the search interval for k*. One can then use Consensus in the narrowed interval. Although it may seem paradoxical, despite its precision performance, FOM does not seem to be competitive in this scenario. Indeed, it is only marginally better than the best performing of WCSS and G-Gap but at least an order of magnitude slower in time.

When one does not account for time, Consensus seems to be the clear winner since it offers good precision performance accross algorithms at virtually the same price in terms of time performance.

## Conclusion

Prior to this research, all the measures we have considered here were perceived as adequate for inferring the number of clusters in a dataset, in particular for gene expression data. This study provides further insights into the relative merits of each of the measures we have considered, from which more accurate and useful guidelines for their use can be inferred. Moreover, extensions and approximations of those measures have also been proposed and they turn out to be competitive, both in time and precision. We have also offered a comparison among three resampling-based prediction methods that is not available elsewhere in the literature.

Overall, Consensus results to be the method of choice, although the fast algorithms may be of great help in limiting the search interval for k*.

It is also to be stressed that no measure performed well on large datasets. In view of this finding, data reduction techniques such as filtering and dimensionality reduction become even more important for class discovery in microarray data. Another promising avenue of research is to design fast approximation algorithms for the computation of the slowest measures, in particular Consensus. Finally, we remark that Gap, Clest, ME and Consensus have various parameters that a user needs to specify. Those choices may affect both time performance and precision. Yet, no experimental study, addressing the issue of parameter selection for those methods, seems to be available in the literature.

## Methods

### Internal measures

#### WCSS and its approximations

Consider a set of *n *items *G *= {*g*_1_,..., *g*_*n*_}, where *g*_*i *_is specified by *m *numeric values, referred to as features or conditions, 1 ≤ *i *≤ *n*. That is, each *g*_*i *_is an element in *m*-dimensional space. Let *D *be the corresponding *n *× *m *data matrix, let *C *= {*c*_1_,..., *c*_*k*_} be a clustering solution for *G *produced by a clustering algorithm and let

(1) $$D_{r}=\sum_{j\in c_{r}}||g_{j}-\bar{g_{r}}|{|}^{2}$$

where $\bar{g_{r}}$ is the centroid of cluster *c*_*r*_. Then, we have:

(2) $$\text{WCSS}(k)=\sum_{r=1}^{k}D_{r}$$

**Figure 2:**
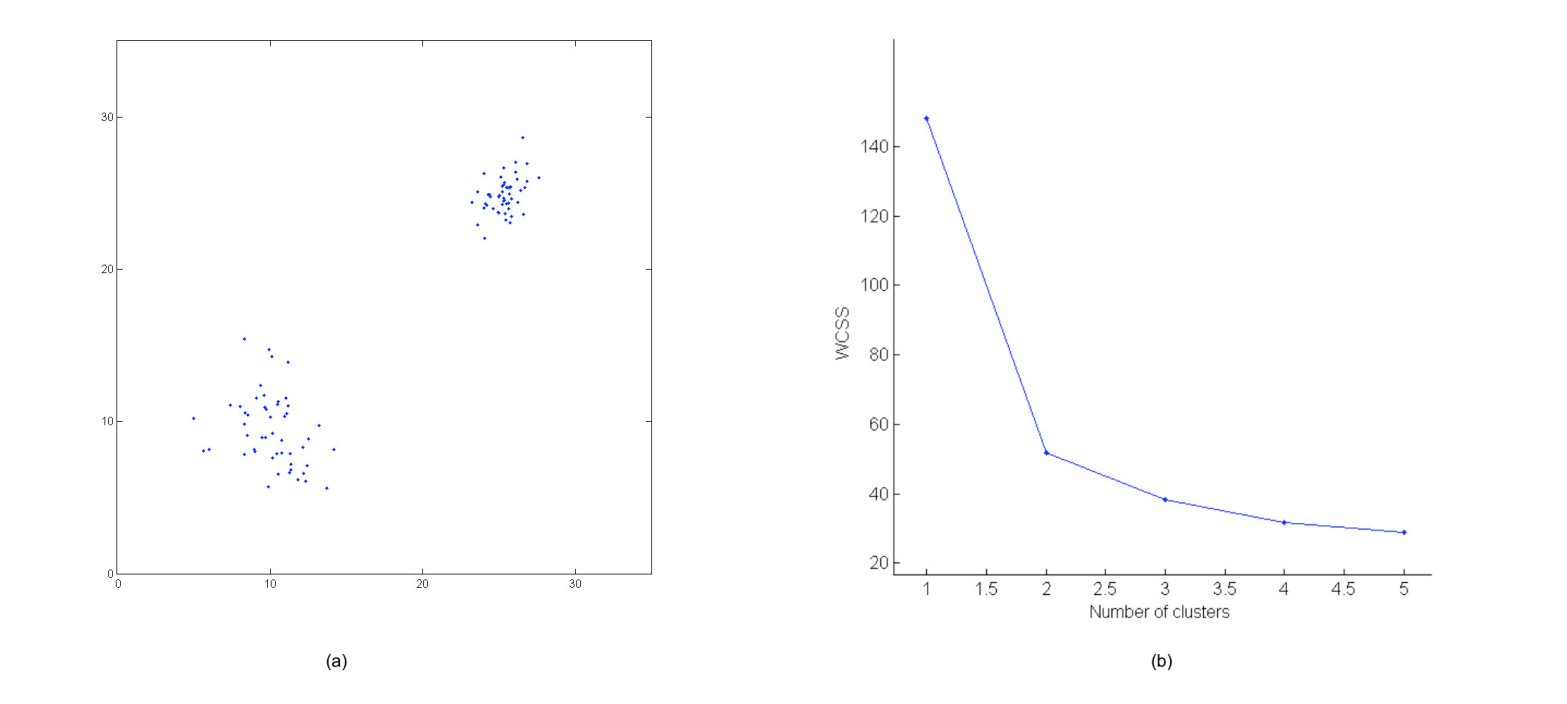
**An example of number of cluster prediction with the use of WCSS**. (a) Dataset. (b) Plot of the values of WCSS with the use of K-means-R on the dataset displayed in (a). The "knee" in the plot indicates the correct number of clusters in the dataset: k* = 2.

Assume now to have *k*_*max *_clustering solutions, each with a number of clusters in [1, *k*_*max*_]. Assume also that we want to use WCSS to estimate, based on those solutions, what is the real number k* of clusters in our dataset. Intuitively, for values of *k *<k*, the value of WCSS should be substantially decreasing, as a function of the number of clusters *k*. Indeed, as we get closer and closer to the real number of clusters in the data, the compactness of each cluster should substantially increase, causing a substantial decrease in WCSS. On the other hand, for values of k* > *k*, the compactness of the clusters will not increase as much, causing the value of WCSS not to decrease as much. The following heuristic approach comes out [15]: Plot the values of WCSS, computed on the given clustering solutions, in the range [1, *k*_*max*_]; choose as k* the abscissa closest to the "knee" in the WCSS curve. Fig. 2 provides an example. Indeed, the dataset in Fig. 2(a) has two natural clusters and the "knee" in the plot of the WCSS curve in Fig. 2(b) indicates k* = 2.

The approximation of the WCSS curve proposed here is based on the idea of reducing the number of executions by a clustering algorithm *A *for the computation of WCSS(*k*), for each *k *in a given interval [1, *k*_*max*_]. In fact, given an integer *R *> 0, the approximate algorithm to compute WCSS uses algorithm *A *to obtain a clustering solution with *k *clusters only for values of *k *multiples of *R*. We refer to *R *as the refresh step. For all other *k*'s, a clustering solution is obtained by merging two clusters in a chosen clustering solution already available. The procedure below gives the high level details. It takes as input *R*, *A*, *D *and *k*_*max*_. Algorithm *A *must be able to take as input a clustering solution with *k *clusters and refine it to give as output a clustering solution with the same number of clusters.

Procedure WCSS-R(*R*, *A*, *D*, *k*_*max*_)

(1) Compute a clustering solution $P_{k_{max}}$ with *k*_*max *_clusters using algorithm *A *on dataset *D*. Compute WCSS(*k*_*max*_) using $P_{k_{max}}$.

(2) For *k *:= *k*_*max *_- 1 down to *k *= 1, execute steps (2.a) (2.b) and (2.c).

(2.a) (Merge) Merge the two clusters in *P*_*k*+1 _with minimum Euclidean distance between centroids to obtain a temporary clustering solution ${P^{\prime }}_{k}$ with *k *clusters.

(2.b) (Refresh) If (*R *= 0) or (*k *mod *R *> 0) set *P*_*k *_equal to ${P^{\prime }}_{k}$. Else compute new *P*_*k *_based on ${P^{\prime }}_{k}$. That is, ${P^{\prime }}_{k}$ is given as input to *A*, as an initial partition of *D *in *k *clusters, and *P*_*k *_is the result of that computation.

(2.c) Compute WCSS(*k*) using *P*_*k*_.

Technically, the main idea in the approximation scheme is to interleave the execution of a partitional clustering algorithm *A *with a merge step typical of agglomerative clustering. The gain in speed is realized by having a fast merge step, based on *k *+ 1 clusters, to obtain *k *clusters instead of a new full fledged computation, starting from scratch, of the algorithm *A *to obtain the same number of clusters. The approximation scheme would work also for hierarchical algorithms, provided that they comply with the requirement that, given in input a dataset, they will return a partition into *k *groups. However, in this circumstance, the approximation scheme would be a nearly exact replica of the hierarchical algorithm. In conclusion, we have proposed a general approximation scheme, where the gain is realized when the merge step is faster than a complete computation of a clustering algorithm *A*. We have experimented with K-means-R and with values of the refresh step *R *= 0, 2, 5, i.e., the partitional clustering algorithm is used only once, every two and five steps, respectively.

### KL

KL is based on WCSS, but it is automatic, i.e., a numeric value for k* is returned. Let

(3) *DIFF*(*k*) = (*k *- 1)^2/*m*^WCSS(*k *- 1) - *k*^2/*m*^WCSS(*k*)

Recall from the previous subsection the behavior of WCSS, as a function of *k *and with respect to k*. Based of those considerations, we expect the following behavior for *DIFF*(*k*):

(i) for *k *<k*, both *DIFF*(*k*) and *DIFF*(*k *+ 1) should be large positive values.

(ii) for *k *> k*, both *DIFF*(*k*) and *DIFF*(*k *+ 1) should be small values, and one or both might be negative.

(iii) for *k *= k*, *DIFF*(*k*) should be large positive, but *DIFF*(*k *+ 1) should be relatively small (might be negative).

Based on these considerations, Krzanowski and Lai proposed to choose the estimate on the number of clusters as the *k *maximizing:

(4) $$KL(k)=|\frac{DIFF(k)}{DIFF(k+1)}|$$

That is,

(5) $$k*=\arg max_{2\leq k\leq k_{max}}KL(k)$$

Notice that *KL*(*k*) is not defined for the important special case of *k *= 1, i.e., no cluster structure in the data.

### Gap and its geometric approximation

The measures presented so far are either useless or not defined for the important special case *k *= 1. Tibshirani et al. [15] brilliantly combine techniques of hypothesis testing in statistics with the WCSS heuristic, to obtain Gap, a measure that can deal also with the case *k *= 1. In order to describe the method, we need to recall briefly null models used to test for the hypothesis of no cluster structure in a dataset, i.e., the null hypothesis. We limit ourselves to introduce the two that find common use in microarray data analysis [15], in addition to another closely related and classic one [34]:

(M.1) Ps (*The Poisson Model*). The items can be represented by points that are randomly drawn from a region *R *in *m*-dimensional space. In order to use this model, one needs to specify the region within which the points are to be uniformly distributed. The simplest regions that have been considered are the *m*-dimensional hypercube and hypersphere enclosing the points specified by the matrix *D*. Other possibilities, in order to make the model more data-dependent, is to choose the convex hull enclosing the points specified by *D*.

(M.2) Pc (*The Poisson Model Aligned with Principal Components of the Data*). This is basically as (M.1), except that the region *R *is a hypercube aligned with the principal components of the data matrix *D*.

In detail, assume that the columns of *D *have mean zero and let *D *= *UXV*^*T *^be its singular value decomposition. Transform via *D*' = *DV*. Now, use *D*' as in (M.1) with sampling in a hypercube *R *to obtain a data set *Z*'. Back transform via *Z *= *Z'V*^*T *^to obtain a new dataset.

(M.3) Pr (*The Permutational Model*). Given the data matrix *D*, one obtains a new data matrix *D*' by randomly permuting the elements within the rows and/or the columns of *D*. In order to properly implement this model, care must be taken in specifying a proper permutation for the data since some similarity and distance functions may be insensitive to permutations of coordinates within a point.

That is, although *D*' is a random permutation of *D*, it may happen that the distance or similarity among the points in *D*' is the same as in *D*, resulting in indistinguishable datasets for clustering algorithms.

**Figure 3:**
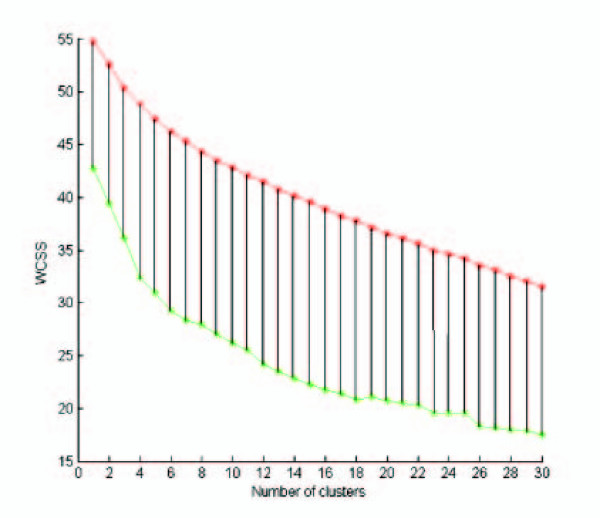
**A geometric interpretation of Gap**. The curve in green is the WCSS computed with K-means-R on the CNS Rat dataset. The curve in red is the *average *WCSS curve, computed on ten datasets generated from the original data via the Ps null model. The vertical lines indicate the gap between the null model curves and the real curve. Since WCSS is expected to decrease sharply up to k*, on the real dataset, while it has a nearly constant slope on the null model datasets, the length of the vertical segments is expected to increase up to k* and then to decrease. In fact, we get k* = 7, a value very close to the number of classes (six) in the dataset.

The intuition behind Gap is brilliantly elegant. Recall that the "knee" in the WCSS curve can be used to predict k*. Unfortunately, the localization of such a point may be subjective. Now, consider the WCSS curves in Fig. 3. That is, the plot is obtained with use of the WCSS(k) values. The curve in green is the WCSS computed with K-means-R on the CNS Rat dataset. The curve in red is the *average *WCSS curve, computed on ten datasets generated from the original data via the Ps null model. As it is evident from the figure, the red curve has a nearly constant slope: an expected behavior on datasets with no cluster structure in them. The vertical lines indicate the gap between the null model curves and the real curve. Since WCSS is expected to decrease sharply up to k*, on the real dataset, while it has a nearly constant slope on the null model datasets, the length of the vertical segments is expected to increase up to k* and then to decrease. In fact, in Fig. 3, we see that k* = 7, a value very close to the number of classes (six) in the dataset. Normalizing the WCSS curves via logs and accounting also for the simulation error, such an intuition can be given under the form of the procedure reported next, where ℓ is the number of simulation steps and the remaining parameters are as in procedure WCSS-R.

Procedure GP(ℓ, *A*, *D*, *k*_*max*_)

(1) For 1 ≤ *i *≤ ℓ, compute a new data matrix *D*_*i*_, using the chosen null model. Let *D*_0 _denote the original data matrix.

(1.a) For 0 ≤ *i *≤ ℓ and 1 ≤ *k *≤ *k*_*max*_, compute a clustering solution *P*_*i*,*k *_on *D*_*i *_using algorithm *A*.

(2) For 0 ≤ *i *≤ ℓ and 1 ≤ *k *≤ *k*_*max*_, compute log(WCSS(*k*)) on *P*_*i*,*k *_and store the result in matrix *SL*[*i*, *k*].

(2.a) For 1 ≤ *k *≤ *k*_*max*_, compute $\text{Gap}(k)=\frac{1}{\ell }\sum_{i=1}^{\ell }SL[i,k]-SL[0,k]$.

(2.b) For 1 ≤ *k *≤ *k*_*max*_, compute the standard deviation *sd*(*k*) of the set of numbers {*SL*[1, *k*],... *SL*[ℓ, *k *]} and let $s(k)=(\sqrt{1+\frac{1}{\ell }})sd(k)$.

(3) Return as k* the first value of *k *such that Gap(*k*) ≤ Gap(*k *+ 1) - *s*(*k *+ 1).

The prediction of k* is based on running a certain number of times the procedure GP taking then the most frequent outcome as the prediction. We also point out that further improvements and generalizations of Gap have been proposed in [30].

**Figure 4:**
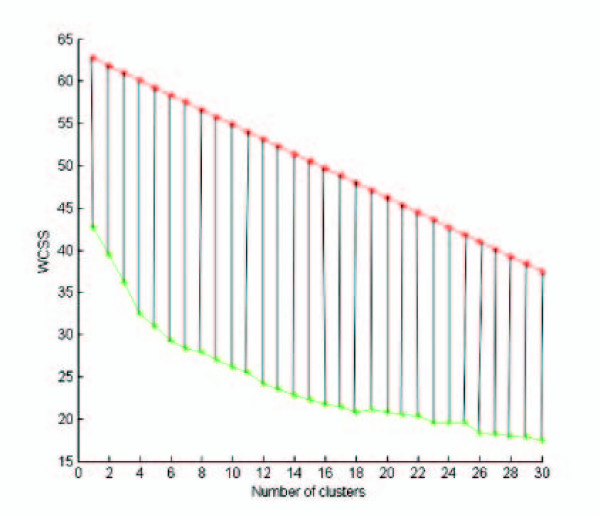
**An example of number of cluster prediction with the use of G-Gap**. The G-Gap Heuristic. The curve in green is a WCSS curve obtained on the CNS Rat dataset with the use of the K-means algorithm. The line in red is obtained by projecting upward the end points of the WCSS curve by *a *units and then joining them. It is a heuristic approximation of WCSS for a null model. The vertical lines have the same role as in Gap and the rule to identify k* is the same, yielding a value k* = 7, a value very close to the correct number of classes(six) in the dataset.

The geometric interpretation of Gap and the behavior of the WCSS curve accross null models suggests the fast approximation G-Gap. The intuition, based on experimental observations, is that one can skip the entire simulation phase of Gap, without compromising too much the accuracy of the prediction of k*. Indeed, based on the WCSS curve, the plot of the log WCSS curve one expects, for a given clustering algorithm and null model, is a straight line with a slope somewhat analogous to that of the log WCSS curve and dominating it. Therefore, one can simply identify the "knee" in the WCSS by translating the end-points of the log WCSS curve on the original dataset by a given amount *a*, to obtain two points *g*_*s *_and *g*_*e*_. Those two points are then joined by a straight line, which is used to replace the null model curve to compute the segment lengths used to predict k*, i.e, the first maximum among those segment lenghts as *k *increases. An example is provided in Fig. 4 with the WCSS curve of Fig. 3. The prediction is k* = 7, which is very close to the correct k* = 6. We point out that the use of the WCSS curve in the figure is to make clear the behavior of the segment lengths, which would be unnoticeable with the log WCSS curve, although the result would be the same.

### Clest

Clest estimates k* by iterating the following: randomly partition the original dataset in a *learning *set and *training *set. The learning set is used to build a classifier C for the data, then to be used to derive "gold standard" partitions of the training set. That is, the classifier is assumed to be a reliable model for the data. It is then used to assess the quality of the partitions of the training set obtained by a given clustering algorithm. Clest generalizes in many respects an approach proposed by Breckenridge [35] and can be regarded as a clever combination of hypothesis testing and resampling techniques. We detail it in the following procedure. With reference to GP, the four new parameters it takes as input are: (a) an external index *E*, i.e., a function that quantifies how similar two partitions are; (b) *p*_*max*_, a "significance level" threshold; (c) *d*_*min*_, a minimum allowed difference between "computed and expected" values; (d) *H*, the number of resampling steps; (e) a classifier C used to obtain the "gold standard" partitions of the training set.

$$Procedure\;Clest(\ell ,H,A,E,D,C,k_{max},p_{max},d_{min})$$

(1) For 2 ≤ *k *≤ *k*_*max*_, perform steps (1.a)-(1.d).

(1.a) For 1 ≤ *h *≤ *H*, split the input dataset in *L *and *T*, the learning and training sets, respectively.

Cluster the elements of *L *in *k *clusters using algorithm *A *and build a classifier C. Apply C to *T *in order to obtain a "gold solution" *GS*_*k*_. Cluster the elements of *T *in *k *groups *GA*_*k *_using algorithm *A*.

Let *SIM*[*k*, *h*] = *E*(*GS*_*k*_, *GA*_*k*_).

(1.b) Compute the observed similarity statistic *t*_*k *_= *median*(*SIM*[*k*, 1],..., *SIM*[*k*, *H*]).

(1.c) For 1 ≤ *b *≤ ℓ, generate (via a null model), a data matrix *D*^(*b*)^, and repeat steps **(1.a) **and **(1.b) **on *D*^(*b*)^.

(1.d) Compute the average of these *H *statistics, and denote it with $t_{k}^{0}$. Finally, compute the p-value *p*_*k *_of *t*_*k *_and let *d*_*k *_= *t*_*k *_- $t_{k}^{0}$ be the difference between the statistic observed and its estimate expected value.

(2) Define a set *K *= {2 ≤ *k *≤ *k*_*max *_: *p*_*k *_≤ *p*_*max *_and *d*_*k *_≥ *d*_*min*_}

(3) Based on *K *return a prediction for k* as: if *K *is empty then k* = 1, else k* = *argmax *_*k *∈ *K *_*d*_*k*_

### ME

Let *f *denote a sampling ratio, i.e., the percentage of items one extracts from sampling a given dataset. The idea supporting ME is that the inherent structure in a dataset has been identified once one finds a *k *such that partitions into *k *clusters produced by a clustering algorithm are similar, when obtained by repeatedly subsampling the dataset. This idea can be formalized by the following algorithm, that takes as input parameters analogous to Clest except for *f *and *Subs*, this latter being a subsampling scheme that extracts *f *percentage items from *D*. It returns as output an (*k*_*max *_- 1) × *H *array *SIM*, analogous to the one computed by Clest and whose role here is best explained once that the procedure is presented.

Procedure ME(*f*, *H*, *A*, *E*, *D*, *Subs*, *k*_*max*_)

(1) For 2 ≤ *k *≤ *k*_*max *_and 1 ≤ *h *≤ *H*, perform steps (1.a)-(1.c).

(1.a) Compute two subsamples *D*^(1) ^and *D*^(2) ^of *D*, via *Subs*.

(1.b) For each *D*^(*i*)^, compute a clustering solution *GA*_*k*,*i *_with *k *clusters, using algorithm *A*, 1 ≤ *i *≤ 2.

(1.c) Let $G{A^{\prime }}_{k,1}$ be *GA*_*k*,1_, but restricted to the elements common to *D*^(1) ^and *D*^(2)^. Let $G{A^{\prime }}_{k,2}$ be defined analogously. Let *SIM*[*k*, *h*] = *E*($G{A^{\prime }}_{k,1}$, $G{A^{\prime }}_{k,2}$).

**Figure 5:**
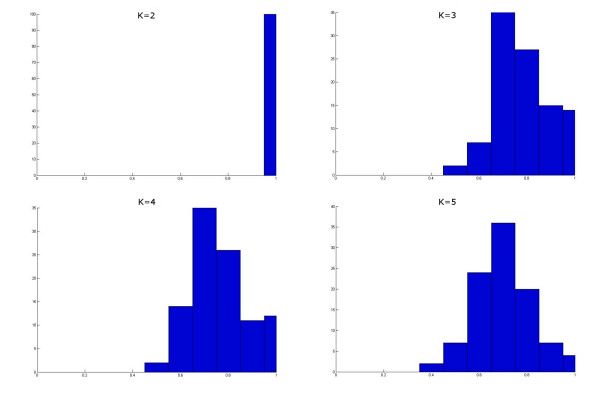
**An example of number of cluster prediction with the use of ME**. Consider the computation of the ME algorithm on the dataset of Fig. 2(a). The histograms plotting the *SIM *values distribution for increasing values of *k *are shown above. The transition allowing for the prediction of k* takes place at *k *= 2 for a correct prediction of k* = 2.

Once that the *SIM *array is computed, its values are histogrammed, separately for each value of *k*, i.e., by rows. The optimal number of clusters is predicted to be the lowest value of *k *such that there is a transition of the *SIM *value distribution from being close to one to a wider range of values. An example is given in Fig. 5, where the transition described above takes place at *k *= 2 for a correct prediction of k* = 2.

### Consensus

Consensus is also based on resampling techniques. In analogy with WCSS, one needs to analyze a suitably defined curve in order to find k*. It is best presented as a procedure. With reference to GP, the two new parameters it takes as input are a resampling scheme *Sub*, i.e., a way to sample from *D *to build a new data matrix, and the number *H *of resampling iterations.

Procedure Consensus(*Sub*, *H*, *A*, *D*, *k*_*max*_)

(1) For 2 ≤ *k *≤ *k*_*max*_, initialize to empty the set *M *of connectivity matrices and perform steps (1.a) and (1.b).

(1.a) For 1 ≤ *h *≤ *H*, compute a perturbed data matrix *D*^(*h*) ^using resampling scheme *Sub*; cluster the elements in *k *clusters using algorithm *A *and *D*^(*h*)^. Compute a connectivity matrix *M*^(*h*) ^and insert it into *M*.

(1.b) Based on the connectivity matrices in *M*, compute a consensus matrix ${ℳ}^{\left(k\right)}$.

(2) Based on the *k*_*max *_- 1 consensus matrices, return a prediction for k*.

The resampling scheme in this case extracts, uniformly and at random, a given percentage of the rows of *D*. As for the connectivity matrix *M*^(*h*)^, one has *M*^(*h*)^(*i*, *j*) = 1 if items *i *and *j *are in the same cluster and zero otherwise. Moreover, we also need to define an indicator matrix *I*^(*h*) ^such that *I*^(*h*)^(*i*, *j*) = 1 if items *i *and *j *are both in *D*^(*h*)^and it is zero otherwise. Then, the consensus matrix ${ℳ}^{\left(k\right)}$ is defined as a properly normalized sum of all connectivity matrices in all perturbed datasets:

(6) $${ℳ}^{\left(k\right)}=\frac{\sum_{h}M^{\left(h\right)}}{\sum_{h}I^{\left(h\right)}}$$

Based on ${ℳ}^{\left(k\right)}$, Monti et al. define a value *A*(*k*) measuring the level of stability in cluster assignments, as reflected by the consensus matrix. Formally,

$$A^{\left(k\right)}=\sum_{i=2}^{n}\left[x_{i}-x_{i-1}]CDF(x_{i}\right)$$

where *CDF *is the empirical cumulative distribution defined over the range [0, 1] as follows:

$$CDF(c)=\frac{\sum_{i<j}l\{M(i,j)\leq c\}}{N(N-1)/2}$$

with *l *equal to 1 if the condition is true and 0 otherwise. Finally, based on *A*(*k*), one can define:

$$\Delta (k)=\{\begin{array}{ll} A(k) & k=2\\ \frac{A(k+1)-A(k)}{A(k)} & k>2 \end{array}$$

Moreover, Monti et al. suggested the use of the function Δ' for non-hierarchical algorithms. It is defined as Δ although one uses *A*'(*k*) = max_*k*' ∈ [2, *k*] _*A*(*k*'). Based on the Δ or Δ' curve, the value of k* is obtained using the following intuitive idea, based also on experimetal observations.

(i) For each *k *≤ k*, the area *A*(*k*) markedly increases. This results in an analogous pronounced decrease of the Δ and Δ' curves.

(ii) For *k *> k*, the area *A*(*k*) has no meaningful increases. This results in a stable plot of the Δ and Δ' curves.

**Figure 6:**
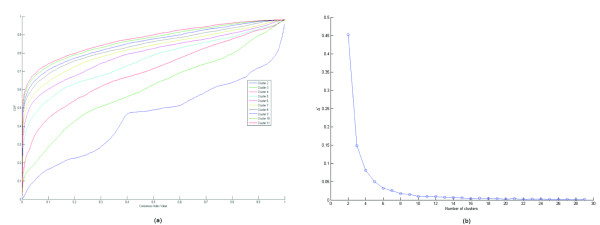
**An example of number of cluster prediction with the use of Consensus**. The experiment is derived from the CNS Rat dataset, with use of the K-means-R clustering algorithm. The plots of the *CDF *curves is shown in (a), yielding a monotonically increasing value of *A *as a function of *k*. The plot of the Δ' curve is shown in (b), where the flattening effect corresponding to k* is evident for *k *≥ k* = 6.

From this behaviour, the "rule of thumb" to identify k* is: take as k* the abscissa corresponding to the smallest non-negative value where the curve starts to stabilize; that is, no big variation in the curve takes place from that point on. An example is given in Fig. 6.

As pointed out in the previous section, our recommendation to use the Δ curve instead if the Δ' curve for non-hierarchical algorithms contradicts the recommendation by Monti et al. The reason is the following: *A*(*k*) is a value that is expected to behaves like a non-decreasing function of *k*, for hierarchical algorithms. Therefore Δ(*k*) would be expected to be positive or, when negative, not too far from zero. Such a monotonicity of *A*(*k*) is not expected for non-hierarchical algorithms. Therefore, another definition of Δ is needed to ensure a behavior of this function analogous to the hierarchical algorithms. We find that, for the partitional algorithms we have used, *A*(*k*) displays nearly the same monotonicity properties of the hierarchical algorithms. The end result is that the same definition of Δ can be used for both types of algorithms. To the best of our knowledge, Monti et al. defined the function Δ', but their experimentation was limited to hierarchical algorithms.

### FOM and its extensions and approximations

FOM is a family of internal validation measures introduced by Ka Yee Yeung et al. specifically for microarray data and later extended in several directions by Datta and Datta [36]. Such a family is based on the jackknife approach and it has been designed for use as a relative measure assessing the predictive power of a clustering algorithm, i.e., its ability to predict the correct number of clusters in a dataset. Experiments by Ka Yee Yeung et al. show that the FOM family of measures satisfies the following properties, with a good degree of accuracy. For a given clustering algorithm, it has a low value in correspondence with the number of clusters that are really present in the data. Moreover, when comparing clustering algorithms for a given number of clusters *k*, the lower the value of FOM for a given algorithm, the better its predictive power. We now review this work, using the 2-norm FOM, which is the most used instance in the FOM family.

Assume that a clustering algorithm is given the data matrix *D *with column *e *excluded. Assume also that, with that reduced dataset, the algorithm produces a set of *k *clusters *C *= {*c*_1_,..., *c*_*k*_}. Let *D*(*g*, *e*) be the expression level of gene *g *and *m*_*i*_(*e*) be the average expression level of condition *e *for genes in cluster *c*_*i*_.

The 2-norm FOM with respect to *k *clusters and condition *e *is defined as:

(7) $$\text{FOM}(e,k)=\sqrt{\frac{1}{n}\sum_{i=1}^{k}\sum_{x\in c_{i}}{\left(D(x,e)-m_{i}(e)\right)}^{2}}$$

Notice that FOM(*e*, *k*) is essentially a root mean square deviation. The aggregate 2-norm FOM for *k *clusters is then:

(8) $$\text{FOM}(k)=\sum_{e=1}^{m}\text{FOM}(e,k).$$

Both formulae (7) and (8) can be used to measure the predictive power of an algorithm. The first gives us more flexibility, since we can pick any condition, while the second gives us a total estimate over all conditions. So far, (8) is the formula mostly used in the literature. Moreover, since the experimental studies conducted by Ka Yee Yeung et al. show that FOM(*k*) behaves as a decreasing function of *k*, an adjustment factor has been introduced to properly compare clustering solutions with different numbers of clusters. A theoretical analysis by Ka Yee Yeung et al. provides the following adjustment factor:

(9) $$\sqrt{\frac{n-k}{n}}.$$

When (9) divides (7), we refer to (7) and (8) as *adjusted *FOMs. We use the adjusted aggregate FOM for our experiments and, for brevity, we refer to it simply as FOM.

**Figure 7:**
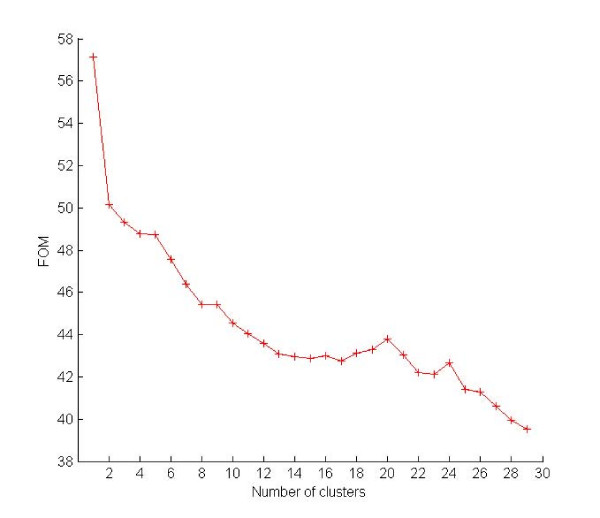
**An example of number of cluster prediction with the use of FOM**. The FOM curve computed on the Leukemia dataset with K-means-R. As for WCSS, the "knee" in the plot indicates the correct number of clusters in the dataset: k* = 3.

The use of FOM in order to establish how many clusters are present in the data follows the same heuristic methodology outlined for WCSS, i.e., one tries to identify the "knee" in the FOM plot as a function of the number of clusters. An example is provided in Fig. 7. Such an analogy between FOM and WCSS immediately suggest to extend some of the knowledge available about WCSS to FOM, as follows:

• The approximation of FOM is based on exactly the same ideas and schemes presented for the approximation of WCSS. Indeed, FOM(*e*, *k*) in equation (7) can be approximated in exactly the same way as WCSS(*k*). Then, one uses equation (8) to approximate FOM. We denote those approximations as FOM-R.

• The G-Gap idea can be extended verbatim to FOM to make it automatic and to obtain G-FOM.

• The KL technique can be extended to FOM, although the extension is subtle. Indeed, a verbatim extension of it would yield poor results (experiments not shown). Rather, consider formula (3), with WCSS(*k*) substituted by FOM(*k*). As *k *increases towards k*, *DIFF*(*k*) increases to decrease sharply and then assume nearly constant values as it moves away from k*. Fig. 7 provides a small example of this behavior. So, one can take as k* the abscissa corresponding to the maximum of *DIFF*(*k*) in the interval [3, *k*_*max*_]. We refer to this method as DIFF-FOM.

### Availability

All software and datasets involved in our experimentation are available at the supplementary material web site. The software is given in a jar executable file for a Java Run Time environment. It works for Linux (various versions—see supplementary material web site), Mac OS X and Windows operating systems. Minimal system requirements are specified at the supplementary material web site, together with installation instructions. Moreover, we also provide the binaries of K-means and hierarchical methods.

## Competing interests

The authors declare that they have no competing interests.

## Authors' contributions

All authors participated in the design of the methods and of the related experimental methodology. DS implemented all of the algorithms, with the exception of Clest, Consensus, ME and an initial version of Gap that were implemented by FU. RG coordinated the research and wrote the report. All authors have read and approved the manuscript.
